# Spatial Distribution and Asymmetry of Surface Electromyography on Lumbar Muscles of Soldiers with Chronic Low Back Pain

**DOI:** 10.1155/2020/6946294

**Published:** 2020-10-26

**Authors:** Zengming Hao, Lin Xie, Jian Wang, Zhenhai Hou

**Affiliations:** ^1^Department of Sports Science, College of Education, Zhejiang University, Hangzhou, China; ^2^Department of Orthopaedics, The No. 903 Hospital of PLA Joint Logistic Support Force, Hangzhou, China; ^3^School of Medicine, Hangzhou Normal University, Hangzhou, China; ^4^Center for Psychological Sciences, Zhejiang University, Hangzhou, China

## Abstract

This study investigated spatial distribution and asymmetry of surface electromyography on lumbar muscles during a sustained contraction in soldiers with and without chronic low back pain. Twenty healthy soldiers and twenty chronic low back pain (CLBP) soldiers had performed the Sorensen test with a duration of 60 seconds. The corresponding muscle fatigue, spatial distribution, and the asymmetry of muscle activity over bilateral paraspinal lumbar regions were measured by the high-density surface electromyography (HDsEMG). The paired and independent samples *t*-tests were performed to compare the degree of muscle fatigue and asymmetry. The repeated-measures analyses of variance (ANOVA) were used to compare spatial distribution between groups and muscle fatigue. The baseline characteristics of soldiers between groups were comparable. CLBP soldiers had significantly less muscle fatigue on both sides of erector spinae compared to healthy ones. The spatial distribution was significantly associated with the group factor but independent of muscle fatigue. In addition, the asymmetry of erector spinae activity in the CLBP soldiers was significantly higher than the healthy one. In conclusion, uneven spatial distribution and asymmetry of lumbar muscle activity play significant roles in CLBP patients. The HDsEMG could be used as an objective method in distinguishing the function of the erector spinae between healthy individuals and CLBP patients during 1 min sustained contraction.

## 1. Introduction

Low back pain (LBP) is neither a disease nor a diagnostic entity of any sort. Nonspecific LBP, in which the pathoanatomical cause of the pain cannot be determined, is the most common one. Nonspecific LBP affects people of all ages and is a leading contributor to disease burden worldwide [1, 2]. Compared to the other chronic diseases, LBP is also a leading chronic health problem and occupies the first place among factors forcing elderly workers to retire prematurely and more people out of the workplace [3]. Dysfunction of the erector spinae is found to be associated with chronic LBP (CLBP) [4, 5].

Surface electromyographic (sEMG) is a simple, valuable, noninvasive technique for measuring the activity, activation, and fatigue of muscle over the lumbar spine and has become an objective marker of LBP in patients [6–8]. The high-density surface electromyography (HDsEMG) is the technological advanced EMG [6, 9]. By using an array of closely spaced electrodes organized in a quadrature grid to record a wide muscle area, HDsEMG allows insights into the spatial distribution of the myoelectric intensity of a muscle. The spatial distribution allows monitoring the activation of different muscle regions, which depends on joint position, contraction level, and duration of movement [9]. In addition, this technique can also be applied to evaluate the development of local muscle fatigue and the symmetrical measurements of the median frequency (MF) and the root mean square (RMS) amplitude of the EMG signals during the sustained isometric effort [10, 11].

In patients with LBP, the erector spinae is more rapidly fatigued [12]. Alternating activation between both sides of the lumbar muscles was related to the development of muscle fatigue during sustained sitting [13]. The subjects with more heterogeneous activities and larger shifts in the upper trapezius activity toward the cranial direction could maintain the longer static contraction. The asymmetry activation was found in the dominant upper trapezius muscle which is more fatigue-resistant than the nondominant one [14]. The asymmetry of the erector spinae is often represented by uncompensated RMS imbalance and alternating frequency [11, 13]. The spatial asymmetry of lumber muscles might also play a critical role in LBP.

During sustained contraction, changes in the spatial distribution of muscle activity may be due to control strategies, such as motor unit substitution or recruitment in preferential muscle regions to reduce the muscle fatigue [15, 16]. Tucker et al. found that there was a change in the spatial distribution of erector spinae muscle activity with fatigue [17]. However, the study was performed only on healthy subjects. Abboud et al. presented that compared to patients with LBP, the EMG spatial alternation of erector spinae is bigger during sustained contraction and the sustained duration is also longer in healthy individuals [18]. A previous study has indicated that the alternation in spatial distribution is correlated to contraction duration [19]. Therefore, the change in the spatial distribution of erector spinae may be related to the duration of sustained contraction.

The Sorensen test provides reliable measures to discriminate between subjects with and without nonspecific low back pain [20]. However, the motivation and subjective effort of participants influence the outcomes of Sorensen test frequently [21]. Therefore, the finite time of Sorensen test is feasible to investigate the EMG differences between healthy individuals and LBP patients. Two 1-min Sorensen tests were used to induce muscle fatigue of the multifidus rapidly in LBP patients [22]. Still, it was confirmed by traditional surface EMG, which is susceptible to the inappropriate electrode displacement. In such a short period of test time, it is not clear whether there is a distinct difference of muscle fatigue between healthy and LBP subjects.

Hence, it is important to further investigate the difference between healthy individuals and LBP patients during a sustained contraction with a shorter duration. Therefore, the present study aims to investigate the differences of spatial distribution and asymmetry in erector spinae between healthy individuals and CLBP patients during 1 min sustain contraction by using the high-density, two-dimensional surface EMG.

## 2. Participants and Methods

### 2.1. Participants

A total of 40 soldiers from the Nanjing Military Army Region were enrolled, including age- and total body mass-matched 20 healthy controls and 20 CLBP patients. Each subject was informed of the purpose and potential risk of the study before the written voluntary consent was obtained. The study was approved by the local Medical Ethics Committee.

### 2.2. Inclusion Criteria

The CLBP patients were diagnosed by the orthopedists, and the symptoms have persisted for at least 6 months. The healthy controls were recruited from the same troop who have never experienced low back pain more than 3 days.

### 2.3. Exclusion Criteria

The subjects with neurological deficits, structure abnormality of spine, and heredity vertebral disease were excluded.

### 2.4. Data Collection

Before the experiment, each subject has filled out surveys regarding personal information (e.g., age, height, body mass, dominant side, history of low back pain), visual analog scale (VAS), and Oswestry low back pain disability questionnaire.

### 2.5. Sorensen Test

**Figure 1:**
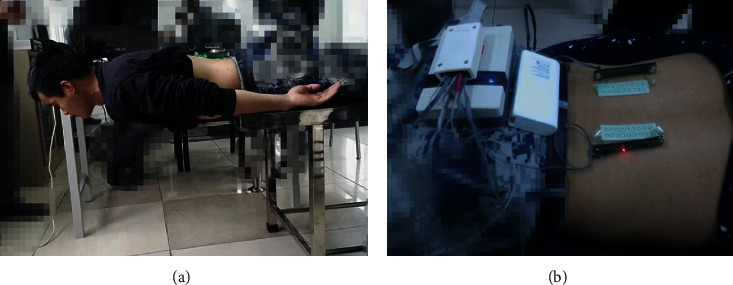
The settings of 1-min Sorensen test. (a) The chronic low back pain patient/healthy control was leaning on the examining table in the prone position with the upper edge of the iliac crests aligned with the edge of the table. (b) The electrode array containing 9*∗*2 electrodes was placed 20 mm away from the spinal cord midline on both sides.

Each subject had received a 1 min Sorensen test. The patient was leaning on the examining table in the prone position with the upper edge of the iliac crests aligned with the edge of the table (as shown in Figure 1(a)). With the arms folded across the chest, each subject was required to maintain the upper body in a horizontal position as possible [23].

### 2.6. Instruments

A noninvasive wearable device was developed to collect HDsEMG. The device consisted of 8 acquisition modules (only two modules were used in this study) and a data fusion module.

A 16-channel matrix-type electrode array was used in each acquisition module to collect sEMG signals. Each acquisition module contained a matrix-type (2  ×  8) electrode array with an interelectrode horizontal distance of 7.5 mm and a vertical distance of 10.05 mm. The signals were sampled at 1000 Hz and filtered at 20–380 Hz band-pass frequency with a 16bit analog-to-digital conversion [24]. HDsEMG signals from these modules were packed in an ARM controller and transferred to a PC via WIFI. The whole device was powered by a rechargeable lithium battery.

### 2.7. Surface EMG Recordings

The surface EMG signals of each side of erector spinae were recorded by electrode arrays, which placed 20 mm away from spinal cord midline [25, 26] (as shown in Figure 1(b)). Surface electromyography (EMG) recordings were obtained bilaterally from the external oblique, rectus abdominis, and L2 and L5 erector spinae. The statistics analysis had revealed that soldiers in both groups had exhibited muscle fatigue on both sides of erector spinae. Surface EMG activities, right vs. left holding times, and decay rate of the median frequency change as the percent changes from the initial value (NMF slope).

### 2.8. Data Processing

The data were processed by Matlab2017b. In total, the 32 differential surface EMG channels were recorded for each patient using 4 electrode arrays (the left and the right-side contains 16 channels, respectively). The root mean square (RMS) and median frequency (MF) were calculated in 500 ms nonoverlapping time-windows. During sustained contraction, the MF of each channel was analyzed by linear regression. The left-side erector spinae muscle (LES) normalized median frequency (NMF) slope and the right-side erector (RES)_NMF slope were represented by the average normalized slope of each channel [13, 22]. The RMS of 16 channels in each side were used to calculated centroid coordinates, modified entropy, and dispersion analyses [18, 19]. In the Sorensen test, the sustained contraction was divided into three stages, including beginning 20 s, middle 20 s, and ending 20 s. The LES_Entropy, LES_CentroidY, LES_DispersionY, RES_Entropy, RES_CentroidY, and RES_DispersionY of each stage were calculated.

### 2.9. Statistical Analysis

Continuous variables were presented as mean and standard deviations (SDs) tested by the Shapiro–Wilks test for the normal distribution. NMF slopes of each group were tested by paired *t*-test. The uncompensated RMS imbalance and alternating frequency were tested by an independent *t*-test. Analyses for the parameters of modified entropy, centroid coordinates, and dispersion were conducted by two-way repeated-measurement analysis of variance (ANOVA). Effect sizes were calculated using Cohen's d values for dependent data. The significance level was set as two-sided *p* < 0.05. All statistical analyses were performed by IBM Statistical Product and Service Solutions (SPSS) statistical software version 22 for Windows (IBM Corp., Armonk, New York, USA).

## 3. Results

### 3.1. Baseline Characteristics

The basic characteristics of the soldiers are shown in Table 1. Both groups were comparable for age, height, body mass, and body mass index (BMI). CLBP soldiers are younger on average (Cohen's *d* = 0.094, *p*=0.83, CLBP vs. Control: 28.6 vs. 29.0 years), the mean of height was higher in CLBP soldiers (Cohen's *d* = 0.977, *p*=0.08, CLBP vs. Control: 176.3 vs. 173.7 cm), the mean of body mass was lower in CLBP soldiers (Cohen's *d* = 0.089, *p*=0.81, CLBP vs. Control: 70.9 vs. 71.7 kg), the mean of BMI was lower in CLBP soldiers (Cohen's *d* = 0.224, *p*=0.49, CLBP vs. Control: 23.0 vs. 23.9 kg/m^2^), but no statistical difference was found in basic characteristics between groups.

### 3.2. Muscle Fatigue

The statistics analysis had revealed that soldiers in both groups had exhibited muscle fatigue on both sides of erector spinae. Compared to the control group, CLBP soldiers had displayed significantly lower (less muscle fatigue) LES_NMF and RES_NMF (LES_NMF slope: −0.11 vs. −0.13, respectively; RES_NMF slope: −0.07 vs. −0.11, respectively) slopes. The RES_NMF slope was significantly lower than the LES_NMF slope in both groups (control group: paired *t*-test *p*=0.001; CLBP soldiers: paired *t*-test *p*=0.002, Table 2).

### 3.3. Spatial Distribution

**Figure 2:**
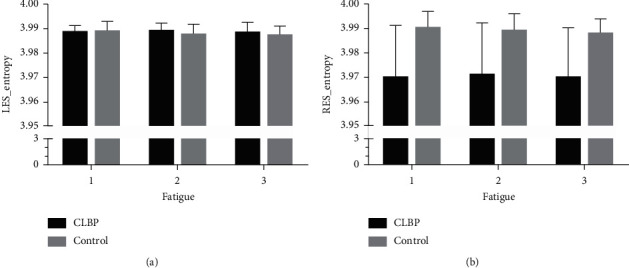
Repeated-measures ANOVA of entropy by fatigue and group factors. ANOVA: analysis of variance; (a) LES: left-side erector spinae muscle; (b) RES: right-side erector spinae muscle; chronic low back pain (CLBP).

**Figure 3:**
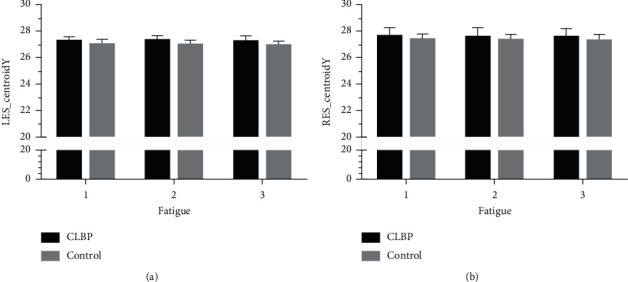
Repeat measures ANOVA of centroid by fatigue and group factors; ANOVA: analysis of variance; (a) LES: left-side erector spinae muscle; (b) RES: right-side erector spinae muscle; CLBP: chronic low back pain.

**Figure 4:**
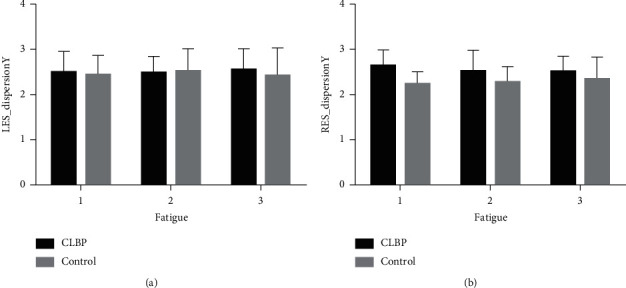
Repeated-measures ANOVA of dispersion by fatigue and group factors. ANOVA: analysis of variance; (a) LES: left-side erector spinae muscle; (b) RES: right-side erector spinae muscle; CLBP: chronic low back pain.

The two-way repeated-measurement ANOVA of entropy, centroid coordinates, and dispersion analyses are shown in Table 3. The results had shown that the group factor was significantly associated with RES_Entropy (*F* = 15.828, *p* < 0.0001), LES_CentroidY (*F* = 6.413, *p*=0.02) and RES_DispersionY (*F* = 14.074, *p*=0.001). In addition, the main effect of fatigue and the association between fatigue and group was not found. The distribution of entropy, centroid, and dispersion by each time stage is shown in Figures 2–4. For RES_Entropy, it was significantly lower in the CLBP group than the control one at each time of sustained contraction (Figure 2(b)). For LES_CentroidY, it was significantly higher in the CLBP group than the control one at each time of sustained contraction (Figure 3(a)). For RES_DispersionY, it was significantly higher in the CLBP group than the control one at each time of sustained contraction (Figure 4(b)).

### 3.4. Asymmetry

The distribution of asymmetry parameters is shown in Table 4. Soldiers in the CLBP group were statistically higher in uncompensated RMS imbalance and alternating frequency as compared to the control group (Cohen's *d* = 2.010, *p* < 0.0001, CLBP vs. Control's uncompensated RMS imbalance: 9.82 vs. 6.21; Cohen's *d* = 1.829, *p* < 0.0001, CLBP vs. Control's alternating frequency: 20.00 vs. 4.10, respectively) (Table 4).

## 4. Discussion

In this study, the groups are similar in terms of anthropometric characteristics. High-density surface EMG was conducted to evaluate muscle fatigue, spatial distribution, and erector spine muscle activity asymmetry in healthy and CLBP soldiers. We had indicated that muscle fatigue had been observed on both sides of erector spinae at the end of the sustained contraction in both groups, and the degree of muscle fatigue was significantly different between the left and the right sides of the erector spine. There were differences in the spatial distribution of erector spinae during sustained contraction between healthy and CLBP soldiers. Asymmetry of erector spine was also observed in both groups, yet the CLBP soldiers had displayed a significantly higher level of asymmetry. Traditional methods such as palpation, anamnesis, and the Borg scale have been used for clinical diagnosis of LBP by a physician or physical therapist [27]. However, these methods relied on the patient's report or the physical therapist's interpretation and involved a great degree of subjectivity [28]. The maximum voluntary isometric contraction (MVIC) is a common measurement technique of muscle strength [29]. However, the lower reliability of MVIC has been reported when assessing trunk muscle fatigue in both healthy controls and LBP patients computationally [30, 31].

The traditional surface EMG, which allows mapping of the MF during a sustained muscle contraction, is commonly used to monitor muscle fatigue. Although this approach is simple and efficient, it requires a long time to complete a complex task identification [32]. The technological advancement of EMG acquisition systems enables the use of HDsEMG [9]. In this study, the HDsEMG signals are applied to assess muscle fatigue and the extent of muscle activation (spatial distribution and asymmetry) during muscle contraction. Differences in muscle fatigue between the CLBP group and the healthy group have been reported [2]. When muscle fatigue occurs during muscle contraction, the NMF usually shifts to lower frequencies [33]. The value of the linear regression slope of NMF is smaller when the stronger fatigue resistance occurs. In the present study, the average value of the NMF slope on the right side of erector spinae was smaller than the left side in both groups. Since all the subjects were right-handed in this study, the right side of the erector spinae was represented as the dominant side. Our results had indicated that fatigue resistance of the dominant side was better than the nondominant side, which is consistent with previous studies [6].

Moreover, the different effects of two sides of erector spinae on muscle fatigue between healthy and LBP groups have also been found [31]. However, unlike the most previous findings [33, 34], we had observed that the fatigue resistance of erector spinae in the healthy soldiers was worse than the CLBP ones regardless of the dominant or nondominant sides. Fatigability in the different parts of the erector spinae is different. Sung et al. had reported neither healthy controls nor LBP patients demonstrated fatigability in the muscles on the dominant side of the back [34]. In this study, we had observed that the healthy group exhibited more fatigability. The variation in the fatigability differences and effect of the dominant side in erector spinae may also be due to patterns of EMG activity being less reliable in initial stages of muscle fatigue [18]. The 60-second sustained contraction used in this study may also induce complicated muscle fatigue of the erector spinae, resulting in the differences of muscle fatigue between our study and previous studies. Entropy represented the degree of homogeneity in muscle activation, and the higher value of entropy indicates that RMS of muscle activity is more evenly distributed. Both centroid and dispersion represented the spatial distribution of muscle activity indirectly.

The higher value of centroid indicates that the spatial distribution of muscle activity has shifted cranially and the higher dispersion represents the more variation in the spatial distribution of muscle activity [19]. In this study, despite significant differences of entropy, centroid, and dispersion during the sustained contraction between two groups, the differences of RES_Entropy, LES_CentroidY, and RES_DispersionY between the CLBP group and the healthy group were found. In addition, levels of uncompensated RMS imbalance and alternating frequency in the CLBP group was statistically higher than those in the healthy group. The distinct differences in spatial distribution between groups were demonstrated in this study. In this study, the entropy of right erector spinae was significantly higher in the healthy group compared to the CLBP group during the sustained contraction, indicating that the dominant erector spinae in the CLBP group displayed higher heterogeneity (enhancement of motor variation). The previous study has reported that the process of sustained contraction is not completely consistent between the dominant and the nondominant sides [18].

We had also found that the spatial distribution of the left and the right sides of erector spinae had undergone some subtle changes during the process. It was worth noting that the spatial distribution of the left side of erector spinae in the CLBP group had shifted cranially more compared to that in the healthy group. This phenomenon might be related to the different roles of the nondominant erector spinae between two groups during the sustained contraction. The spatial distribution variability of the right erector spinae activity in the CLBP group was greater in the cranial-caudal direction compared to that in the healthy group, which is opposite to the results from the previous study [31]. Uncompensated EMG imbalances indicate the global presence of uneven activation behavior across all contralateral muscle sites in the lumbar back [11]. We had found that uncompensated RMS imbalances between the left and right side of the erector spinae in LBP patients and healthy subjects, which may be related to the differences between the dominant and nondominant sides. The absolute value of uncompensated RMS imbalances and alternating frequency were significantly higher in the CLBP patients compared with the healthy subjects, indicating smaller asymmetry in the healthy group. Thus, the presence of a higher asymmetry of erector spinae muscle activity, that is, larger uncompensated RMS imbalances and alternating frequency, may have been an indication of a nonhealthy back muscle function in our patient group. A similar observation was also made by Oddsson and De Luca [11]. The higher level of asymmetry exhibited in the CLBP group may also suggest that the presence of pain caused a redistribution of the activation behavior between synergistic muscles of the lumbar back [11].

Subjects with a larger shift in the spatial distribution of muscle activity could sustain the static contraction longer [18, 19] and the healthy group exhibit longer duration and the larger shift of spatial distribution compared to the CLBP group [2, 31]. Therefore, CLBP patients who suffered from pain do not have to undergo the measurement of their trunk muscle endurance during the long-sustained contraction. We have introduced for the first time the concept of distinguishing CLBP patients from healthy individuals in a very short duration (e.g., 1 min) based on the spatial distribution of muscle activity. Our results had indicated that the spatial distribution of muscle activity had been changed in the CLBP patients during the beginning of a sustained contraction, especially on the dominant side of erector spinae. In addition, the asymmetry of muscle activity of erector spinae is also significantly higher in the CLBP soldiers. However, whether the increase of asymmetry may lead to the change of spatial distribution of muscle activity and vice versa requires further investigation in the future. The study is not without limitations. The duration of the Sorensen test was measured by only 1 min, not compared with the traditional Sorensen test, which is worthy of further research. In addition, the effectiveness of different rehabilitation methods for soldiers with chronic low back pain should be investigated in future studies.

## 5. Conclusion

In this study, the uneven spatial distribution and asymmetry of lumbar muscle activity detected by the HDsEMG play significant roles in CLBP patients. The HDsEMG could be used as an objective and simple method in distinguishing the function of the erector spinae between a healthy individual and CLBP patients during 1-min sustained contraction.

## Figures and Tables

**Table 1:** Basic characteristics of the study population.

	Control (*N* = 20)	CLBP (*N* = 20)	Cohen's *d*^a^	*p* value^b^
	Mean ± SD			
Age, years	29.0 ± 4.6	28.6 ± 3.8	0.094	0.83
Height, cm	173.7 ± 2.9	176.3 ± 2.4	0.977	0.08
Body mass, kg	71.7 ± 9.2	70.9 ± 8.7	0.089	0.81
BMI, kg/m^2^	23.9 ± 3.6	23.0 ± 4.4	0.224	0.49
Pain duration, years	N/A	5.8 ± 4.4	N/A	N/A
Pain side, L/R	N/A	11/9	N/A	N/A
VAS	N/A	4.2 ± 1.5	N/A	N/A
ODI	N/A	16.9 ± 6.3	N/A	N/A

^a^Cohen's *d*= (M2 − M1)/SD_pooled_. ^b^Paired *t*-test. CLBP: chronic low back pain; SD: standard deviation; BMI: body mass index. VAS: visual analog scale; ODI: Oswestry disability index.

**Table 2:** Two-way ANOVA between groups and muscle side.

Group	Control	CLBP	*p* value^a^ (*F*)	*p* value^b^ (*F*)	*p* value^c^ (*F*)
LES (NMF slope, %/min)	−0.13 ± 0.04	−0.11 ± 0.04	<0.0001 (2.334)	0.003 (89.364)	0.512 (0.458)
RES (NMF slope, %/min)	−0.11 ± 0.03	−0.07 ± 0.05			

^a^Two-way ANOVA between control and CLBP;^b^ two-way ANOVA between LES and RES;^c^ two-way ANOVA between interaction term in group and muscle side. Data are shown as mean ± standard deviation. ^*∗*^Statistically significant (*p* < 0.05). CLBP: chronic low back pain; LES: left-side erector spinae muscle; RES: right-side erector spinae muscle; NMF: normalized median frequency.

**Table 3:** Repeat measures ANOVA of entropy, centroid, and dispersion by fatigue and group factors.

		df	*F*	*p* value
LES_Entropy	Group	1	0.464	0.50
	Fatigue	2	3.167	0.05
	Group^*∗*^Fatigue	2	1.935	0.15
RES_Entropy	Group	1	15.828	<0.0001^*∗*^
	Fatigue	2	0.333	0.72
	Group^*∗*^Fatigue	2	0.63	0.54
LES_CentroidY	Group	1	6.413	0.02^*∗*^
	Fatigue	2	0.292	0.71
	Group^*∗*^Fatigue	2	1.580	0.22
RES_CentroidY	Group	1	1.652	0.21
	Fatigue	2	2.463	0.10
	Group^*∗*^Fatigue	2	0.247	0.74
LES_DispersionY	Group	1	0.151	0.70
	Fatigue	2	0.047	0.95
	Group^*∗*^Fatigue	2	0.186	0.83
RES_DispersionY	Group	1	14.074	0.001^*∗*^
	Fatigue	2	0.246	0.78
	Group^*∗*^Fatigue	2	0.954	0.39

LES: left-side erector spinae muscle; RES: right-side erector spinae muscle; Centroid Y: centroid in *y*-axis; Dispersion Y: dispersion in *y*-axis. ANOVA: analysis of variance. ^*∗*^Statistically significant (*p* < 0.05). df: degree of freedom; F: statistic amount of testing.

**Table 4:** The distribution of asymmetry parameters.

	Control (*N* = 20)	CLBP (*N* = 20)	Cohen's *d*^a^	*p*-value^a^
	Mean ± SD			
Uncompensated RMS imbalance	6.21 ± 0.94	9.82 ± 2.36	2.010	<0.0001^*∗*^
Alternating frequency	4.10 ± 2.22	20.00 ± 12.09	1.829	<0.0001^*∗*^

^a^Cohen's *d*= (M2-M1) ⁄ SD_pooled_. ^b^*T*-test. CLBP: chronic low back pain; SD: standard deviation; RMS: root mean square. ^*∗*^Statistically significant (*p* < 0.05).

## Data Availability

The data are available upon reasonable request to the corresponding author.
